# The DaNa^2.0^ Knowledge Base Nanomaterials—An Important Measure Accompanying Nanomaterials Development

**DOI:** 10.3390/nano8040204

**Published:** 2018-03-29

**Authors:** Harald F. Krug, Nils Bohmer, Dana Kühnel, Clarissa Marquardt, Katja Nau, Christoph Steinbach

**Affiliations:** 1 NanoCASE GmbH, St. Gallerstr. 58, 9032 Engelburg, Switzerland; hfk@nanocase.ch; 2 Society for Chemical Engineering and Biotechnology (DECHEMA), Theodor-Heuss-Allee 25, 60486 Frankfurt am Main, Germany; nils.bohmer@dechema.de; 3 Department Bioanalytical Ecotoxicology (BIOTOX), Helmholtz Centre for Environmental Research GmbH—UFZ, Permoserstraße 15, 04318 Leipzig, Germany; dana.kuehnel@ufz.de; 4 Institute for Automation and Applied Informatics (IAI), Karlsruhe Institute of Technology (KIT), Hermann-von-Helmholtz-Platz 1, 76344 Eggenstein-Leopoldshafen, Germany; clarissa.marquardt@kit.edu (C.M.); katja.nau@kit.edu (K.N.)

**Keywords:** nanomaterials, nanotoxicology, safety, basic information, literature criteria checklist

## Abstract

Nanotechnology is closely related to the tailored manufacturing of nanomaterials for a huge variety of applications. However, such applications with newly developed materials are also a reason for concern. The DaNa^2.0^ project provides information and support for these issues on the web in condensed and easy-to-understand wording. Thus, a key challenge in the field of advanced materials safety research is access to correct and reliable studies and validated results. For nanomaterials, there is currently a continuously increasing amount of publications on toxicological issues, but criteria to evaluate the quality of these studies are necessary to use them e.g., for regulatory purposes. DaNa^2.0^ discusses scientific results regarding 26 nanomaterials based on actual literature that has been selected after careful evaluation following a literature criteria checklist. This checklist is publicly available, along with a selection of standardized operating protocols (SOPs) established by different projects. The spectrum of information is rounded off by further articles concerning basics or crosscutting topics in nanosafety research. This article is intended to give an overview on DaNa^2.0^ activities to support reliable toxicity testing and science communication alike.

## 1. Introduction—Nanosafety: An International Issue

Nanotechnology is considered one of the key technologies of the 21st century. The success of this fascinating technology is based on its versatility. During the last decades, nanomaterials have found their way in our personal and professional everyday life, whilst at the same time the awareness of potential risks of these new materials for humans and/or the environment continuously increased during the last ten years. The nanosafety aspect is now a topic around the world. Whereas only five nanosafety projects were funded in the 6th Framework programme of the EU (2002–2006), around 50 projects and initiatives on nanosafety were funded in the 7th EU Framework programme between 2007 and 2013. At the moment (February 2018), 19 projects are running under H2020 (source EU NanoSafety Cluster [1]). All nanosafety-related projects are consolidated in the so-called EU NanoSafety Cluster, an initiative of the European Commission Directorate-General for Research and Innovation (DG RTD). It aims to maximize synergies between research projects on a European or national level addressing all aspects of nanosafety including toxicology, ecotoxicology, exposure assessment, mechanisms of interaction, risk assessment, and standardization. More information is available on www.nanosafetycluster.eu.

Within the first phase (2014–2015) of the European Work Programme “Horizon 2020—Topic Nanotechnologies, Advanced Materials und Production (NMP)”, the nanosafety aspect gained more and more importance. One expected impact of the calls for proposals was “Promoting safe-by-design approaches in collaboration with the EU NanoSafety Cluster and contributing towards the framework of EU nanosafety and regulatory strategies…” [2].

In Germany, the Federal Ministry of Education and Research (BMBF) funds projects on nanosafety, together with industry, to fill knowledge gaps and to initiate measures to identify and minimize risk; it has done this since the beginning of the 1990s. Since 1998, funds made available for nanotechnology within the framework of BMBF project funding have increased by a factor of four. Competence centers were established simultaneously as supporting infrastructures (source BMBF). The BMBF raised the framework programs “Materials Innovations for Industry and Society - WING” (2004-2014) and “From Material to Innovation” (2015–2025) and recently renewed the “Action Plan Nanotechnology 2020” [3]. Within these frameworks, the Ministry funds the initiatives on the topic of “Safe handling of synthetic nanoparticles—Studying the effects on humans and the environment—NanoCare”. DaNa and the follower DaNa^2.0^ are part of this topic and present the funded projects on a website under the column “Projects—Current research”. Moreover, an interactive map for nanosafety sponsorship shows project partners across Europe. Table 1 summarizes examples for national nanosafety initiatives from Europe, as well as from Asia.

In Canada and USA, several national projects and initiatives are running, for example, the National Institute for Nanotechnology (NINT) from The National Research Council of Canada [4] or the NanoPortal as a gateway to the Government of Canada’s information on nanotechnology [5].

In the USA, NIOSH is the leading federal US agency conducting research and providing guidance on the occupational safety and health implications and applications of nanotechnology [6].

The Organisation for Economic Co-operation and Development (OECD) also cares for the safety of manufactured nanomaterials [7].

With the example of nanosafety research in mind, it is obvious that not only chemicals have to be safe or labeled accordingly but also any kind of “material” used for products on the market. Previous incidences demonstrated in a very effective way the necessity of safety research to evaluate potential risks of advanced materials, especially nanomaterials, in a more sustainable and comprehensive manner. Examples from the last century have demonstrated dramatically what happens if this rule is neglected. Although first evidence for health effects and tumor induction was found in the early 1930s, it took 40 years until asbestos has been regulated in Europe by the end of the 1970s [8]. Actually, we have not reached the maximum of death cases induced by asbestos, and still its use is allowed in some countries. To avoid such severe situations for human health and reduce the financial loss during the process of compensation, it is necessary to address safety aspects in a very early stage of development of new materials. However, knowledge of mechanisms of toxicity opens the way to modify chemical compounds to render them non-toxic, often by changing ingrained process steps. For more than 15 years a new philosophy called “green chemistry” [9] has been propagated in labs and production sheds, finally generating the idea of “green toxicology” [10]. One of the major doctrines of green chemistry/green toxicology is “Benign-by-Design”, which means to substitute toxic compounds or to reduce them to a minimum just in the planning phase of a new product. Nevertheless, until that time point when all production processes in the world will introduce such ideas and only safe products will be sold, we have to accept that most of our daily life chemicals and materials contain, to a certain extent, a harmful part and may pose a risk to health and environment during their production, use, or deposition. Nanotechnology and nanomaterials, especially nanoobjects, are discussed to give reason for concern when used in large amounts in a multitude of products [11,12,13]. At the same time, the number of publications presenting data on the toxicology of nanomaterials is tremendously increasing, but most studies are not useful for risk assessment of engineered nanomaterials (ENM) [14,15]. This fact is attributed to poor and inappropriate study design, as published in several studies demonstrating various pitfalls when working with nanomaterials, which hamper the reliability of observed adverse effect [16,17,18,19,20,21,22,23]. Moreover, contaminations with endotoxins or other substances may have larger effects than the nanomaterials themselves [19,24,25,26,27]. Taken together, these considerations on extensive evaluation of published data seem mandatory before conclusions for environment, health, and safety can be drawn. This is one of the integral parts of the project DaNa^2.0^. Here a knowledge base of well-evaluated literature data is presented that offers credible information about nanomaterials in advanced products. The knowledge base presented here is not established to offer information for regulatory processes such as TSCA or REACH applications; the aim of this information platform is to provide well-evaluated literature data that offer credible information about nanomaterials in advanced products for all interested social groups.

## 2. Study Design and Methodology

The DaNa^2.0^ project follows the overall aim of providing a non-biased, quality-approved, and up-to-date knowledge base on all aspects of nanosafety research. It covers the fields of human and environmental toxicology, biology, physics, chemistry, and pharmacy. An interdisciplinary team of experts from different research areas analyses scientific publications, reports, project results, and latest news on human and environmental toxicology. This information is constantly evaluated and transferred into a sophisticated, application-oriented database, which can be accessed via a website (www.nanoobjects.info). The central tool of the database provides a unique link between nanomaterials in real applications (e.g., everyday products or medical products) and their potential impacts/toxicological effect(s), and can be easily accessed by the interested visitor.

To ensure that only results of literature, which comply with a high scientific standard, find their way into the database, the DaNa^2.0^ expert team developed the literature criteria checklist “Methodology for selection of publications” [28]. This list includes the definition of mandatory and desirable assessment criteria that are acknowledged worldwide within the scientific community. These criteria need to be fulfilled in order for the publication to be integrated into the DaNa^2.0^ knowledge base. As this database not only contains evaluated, approved, and commented information, but also various ways of accessing the data information, it rather is a knowledge base than a pure database.

## 3. Knowledge Base—Content and Statistics

### 3.1. Information Platform to Support Sustainable Material Development

As mentioned before, it is absolutely essential for the successful application of any new material or new substance to assess its safety for the workers, the customers, and ultimately the environment. Important factors when dealing with such potential risks are good-quality information sources, which provide the necessary facts without overwhelming the user (material developer, occupational health and safety (OHS) personnel, etc.) and practical guidelines on how to handle and test such new materials. Both factors—good-quality and science-based information on safety issues for nanomaterials, as well as standard operation procedures (SOPs) established and validated within national and international projects, are provided on the DaNa^2.0^ web platform www.nanoobjects.info [13,29].

For any industrial setting, material safety data sheets (MSDS) are an essential necessity and are important to consider when developing new materials for future applications. Within the nanosafety community, it has been well established that the material characterization is a key challenge for any nanosafety assessment and safer-by-design approaches. The physico-chemical material characterization therefore needs to be fit-for-purpose and relevant for intended use and has to fulfill minimal characterization requirements [30,31,32]. The above described “DaNa Literature Criteria Checklist”, which includes such minimal information requirements for both physico-chemical and also biological characterization, is also a good tool to support these requirements.

The “Knowledge Base Nanomaterials” provides in-depth information on human health and environment-related safety aspects of currently 26 nanomaterials, together with material-related information on production and further applications. This knowledge base is complemented by an additional sophisticated database linking 65 market-relevant (nano) applications directly with the respective nanomaterials and potential effects for that particular material-application combination. The applications can be allocated to different sectors such as medical products or electronic goods. This implies a varying likelihood of exposure for human beings and the environment. The higher the likelihood of exposure, the more important it is to consider safety aspects (see next chapter and Appendix A Table A1).

Industry, as well as related OHS personnel, is heavily relying on the manufacturers and suppliers’ information on their respective product to ensure appropriate and safe handling of the raw product, as well as in later stages for the safety of the intermediate or final product. With this in mind, industry has a high responsibility to provide such information on safety measures tailored specifically to nanoscaled materials in combination with the advertisement or their respective products. However, there still seems to be an overall lack of information, which is indicated by the persistently high download rates of MSDS provided on the DaNa^2.0^ website that were generated within the NanoCare project that was finalized 9 years ago.

Nanoscale Titanium dioxide (TiO_2_) is the most prominent representative for this phenomenon. As demonstrated by the high download rates shown in Table 2, this nanomaterial receives a tremendous amount of interest across the different countries. Given the high production volumes of TiO_2_(nano) (40,000 t annually) [33], and the many applications bearing a high exposure potential to humans (pigments, sun screen), there is a high need for information on occupational and consumer health, which, at the moment, seems not to be provided by the manufacturer or supplier in an adequate manner.

### 3.2. Platform Informing on the Applications of Advanced Materials, Its Implications for Consumers, and Safety Issues in an Integrative Way

Nanotechnology is nowadays everywhere, and various nanomaterials are integrated more or less obviously into a broad array of different products and applications. However, for consumers it is often hard to identify which products contain nanomaterials at all, and more specifically what type of nanomaterial, is used in what type of product. What is the intended purpose of these nanomaterials in the products and what benefit do they offer the consumer? In that sense, it is vital to provide the interested visitor with the opportunity of getting specific application-oriented information together with the implications for consumers. Hence, the DaNa^2.0^ knowledge base provides a designated web-tool allowing the visitor an easy way and access to link nanomaterials to specific applications and vice versa [34]. In addition, for each of the 26 nanomaterials included in the knowledge base, in-depth information on material properties, additional applications, and production is provided (Appendix A Table A1 Nanomaterials and their applications as listed in the DaNa^2.0^-Knowledge base).

Depending on the type of application, the likelihood of the consumer getting into contact with nanomaterials differs significantly, which in turn has a major impact on potential positive or negative effects on the consumer. Nanomaterials used, e.g., in sunscreen and pharmaceuticals, will get directly in contact with the consumer, whereas this is very limited, e.g., for those nanomaterials applied in paints as pigments. Direct contact with the nanomaterial might occur during the painting process itself, whereas during the use phase (paint on the wall or façade) the potential contact with humans is very low/negligible. Nanomaterials integrated in products such as solar panels have no direct interaction with neither humans nor the environment. The nanomaterials integration into the product is of particular relevance, as nanomaterials firmly embedded in a matrix remain there and will not be encountered by the consumer.

With regard to the environment, nanomaterial emissions will likewise heavily depend on the type of application and the integration into the product. The nanomaterial production and the end-of-life phase (when nanoproducts end up as waste) are considered as most important for nanomaterials release into different environmental compartments. All these different facets of nanomaterial and application-specific issues for both the consumer and the environment are considered and brought together in the knowledge base.

Based on the starting point of a search, visitors can assess all information either starting from a specific type of nanomaterial or starting from a specific application.

A brief overview on materials properties, applications, and safety issues is provided (one-pager). Detailed articles with graded depth of complexity inform on the origin and production of the nanomaterials, together with the most prominent properties leading to the use of a nanomaterial for a specific purpose.

As the acceptance of novel technologies heavily depends on transparency regarding safety issues, in addition to material properties extensive information on the state of knowledge of nanomaterial safety is provided. This includes reliable information on environmental and human health, linking hazard data to the likelihood of exposure, which can be deduced from the way the consumer gets into contact with the nanomaterial and how the nanomaterial is integrated into the product or application.

**Figure 1:**
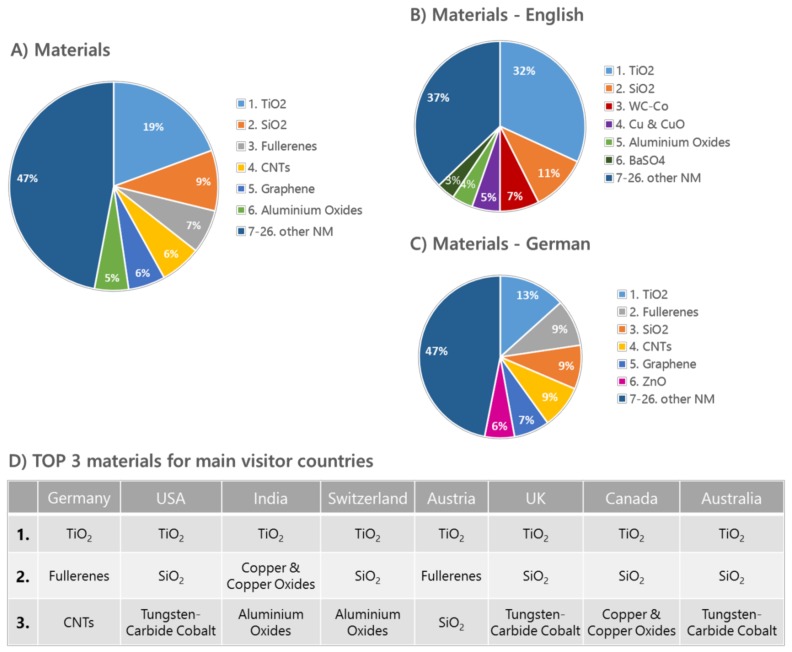
DaNa^2.0^ Website Access Statistics 2016 generated using Webanalytic Tools PIWIK [35] and Google Analytics [36], showing access-data for the most popular six materials. (**A**) Total access of all ENM; access-data sorted by website language in: (**B**) English and (**C**) German. (**D**) shows an overview of the three most accessed nanomaterials 2016 in correlation with the respective visitor countries.

Looking at the popularity of the nanomaterial articles within the knowledge base, a very strong interest in titanium dioxide independent of the articles’ language or the national background of the visitors becomes obvious. The most visited nanomaterials webpages include, besides TiO_2_, also new materials such as fullerenes, graphene, and carbon nanotubes (Figure 1). Differences in visitor interests become more apparent when comparing the access numbers for the two languages German and English. Visitors with an English background seem to favor additionally the nanomaterials tungsten carbide/cobalt, silicon dioxide, and copper/copper oxides, whereas the ranking for the German-based articles is similar to the addition of zinc oxide to the overall visitor interests.

Why certain nanomaterials are favored by various countries is not easy to explain and mostly the result of various factors such as the current public discussion, the focus of the national industry, and research, as well as participation in international projects. Even the native German-speaking countries Austria, Germany, and Switzerland have different nanomaterial priorities, which include, besides TiO_2_ or fullerene, also silicon dioxide, carbon nanotubes, and aluminum oxides. Barium sulphate seems to be of great importance for India, Canada, UK, and Australia, with India being one of the main exporters of barium sulphate in general. In terms of applications that can be found in bone cement or contrast agents, BaSO_4_ is mainly used as reference material for nanosafety research in cell culture or animal testing scenarios.

Tungsten carbide, together with its cobalt-modified variant, shows high access numbers from countries such as the USA, Canada, UK, and Australia. One reason might be a great industrial interest and usage of that material in tools, and the USA and Canada are amongst the main exporters of that particular material.

As most visitors access the website with a specific question in mind, they are offered a selection of the most frequently asked questions (FAQs) and answers addressing the most prominent issues related to nanotechnology [37]. In addition, the website provides the opportunity to directly interact with the DaNa^2.0^ experts via the contact form [38] or via E-Mail.

## 4. Summary

Today the DaNa^2.0^ Knowledge Base is an internationally unique collection of information on material properties, applications, and safety aspects of engineered nanomaterials. To our knowledge, no other database worldwide shows a direct correlation between material properties on the one hand and applications of ENM on the other hand that distinguishes between the application-related potentially different (e.g., toxicological) effects on human health and the environment.

The authors notice an ongoing strong public interest in the theme “safety of nanomaterials” and the responses from certain groups, ranging from large industry, small and medium enterprises (SMEs), up to non-governmental organisations (NGOs). This reflects the high demand on this kind of impartial information, which was and still is being appreciated by all interested stakeholders. For this purpose, the DaNa^2.0^ partners built up a competence pool enabling not only the evaluation of relevant scientific literature in a multidisciplinary field but also for breaking down complex scientific results into compact and simplified content addressing the different needs of the various interest groups.

**Figure 2:**
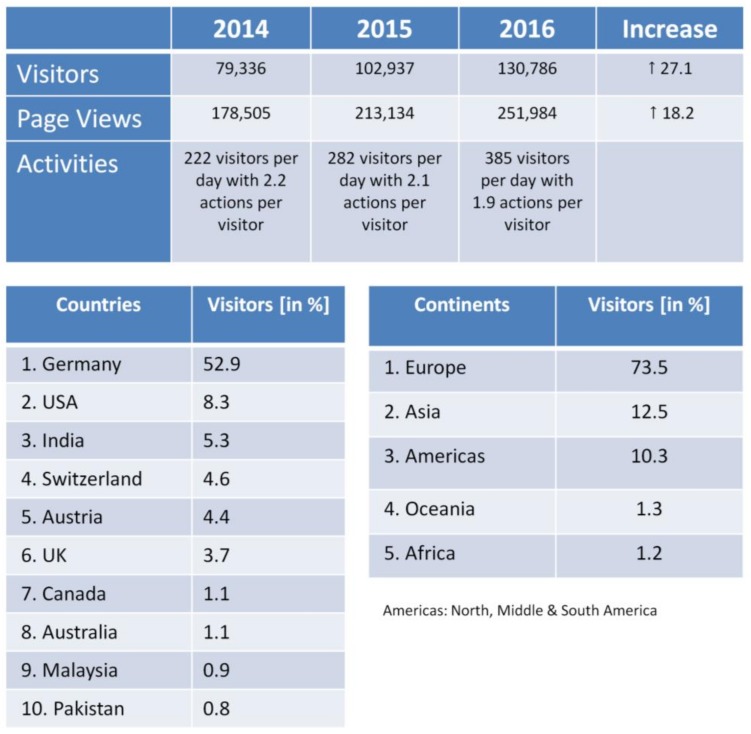
Website Access Statistics 2016 generated using the same tools as for Figure 1, showing the development of visitor numbers & page views from 2014 to 2016 (top); ranking of visitor numbers in 2016 sorted by country and continent of origin.

A strong argument for the success of the DaNa^2.0^ website is the number of visitors that has being constantly increasing over the last few years (Figure 2). In 2015, the visitor number exceeded the 100,000 visitor threshold by reaching an all-time high in 2016 of more than 130,000 visitors per year. This is enormous accomplishment for a scientific website. The majority of the websites’ visitors with about 62% originates from the German speaking countries (Austria, Liechtenstein, Switzerland, and Germany), but numbers for international visitors (38% in 2016) are constantly increasing, with the USA and India being amongst the Top 3 visitor countries or the DaNa^2.0^ website. Another success factor for the website is the positive ranking with search engines like Google. When searching for information on nanomaterials on Google Germany, the DaNa^2.0^ web platform is listed amongst the top 5 to 10 search results for all 26 ENM included in the DaNa^2.0^ Knowledge Base. In addition, the evaluation of the collected webanalytics data suggest a great public interest in the website as indicated by tracking “referrer links”, so-called permanently installed hyperlinks on other websites. The referring websites also include industry, SMEs, as well as various national and international institutions surmounting the area of the scientific nanosafety community at large.

## 5. Conclusions

The freely accessible information and services provided to all interested people is one reason for the high acceptance of the DaNa^2.0^ website. In addition, the website is addressing the issue of lack of practical support for standardized nanotoxicology research being available on the internet. The lab protocols and SOPs published on the DaNa^2.0^ website were generated, and in parts validated, within the context of various national and international research projects, thereby offering a good basis for scientifically profound and—most importantly—reproducible work. As well, the transparent quality assessment procedure of literature along the DaNa^2.0^ criteria catalogue provides valuable support to scientists for designing meaningful experiments. Both assessed literature and the criteria catalogue are made available at the www.nanoobjects.info website.

Unfortunately, financing the website through payments from industry, NGOs, or interested citizens is not a suitable solution as a business model. While the first two groups might be interested in financing the website, the independence and objectivity of the website and its content would drastically lose its credibility with the public. The consumer, on the other hand, will not pay for the kind of information provided on the website. Therefore, the website and thus the DaNa^2.0^ project will have to remain to rely on public funding, on national and international level, respectively.

The dependency on public funding is one of the most challenging obstacles for the DaNa^2.0^ project: on the one hand, this project needs a long run-time, and on the other hand, public financing for long-term projects is often very difficult. The long period of funding is a result of establishing, updating, and maintaining the current website content, which is a huge and time-consuming effort. Therefore, the desired future scientific adaptations and possible additional topics that may be presented on the website must be considered carefully. Furthermore, IT-based tools that support the scientific work might be developed, and actual material-related information could be included, slightly broadening the scope from nanomaterials to new and advanced materials. Nevertheless, the main focus should always be placed on toxicological concerns and/or safety aspects.

## Figures and Tables

**Table 1:** Examples for Nanosafety Initiatives in Europe and Asia.

**Nanosafety Initiatives in Europe**		
**Country**	**Initiative**	**Website**
Denmark	Danish Nano Safety Centre	http://nanosafety.dk
EU	NanoSafetyCluster	https://www.nanosafetycluster.eu/
France	Platform Nanosafety	http://www.nanosafety-platform.com/cea-tech/pns
Sweden	SWETOX	http://swetox.se/en/collaboration/swenanosafe-the-national-platform-for-nanosafety/
Switzerland	InfoNano	https://www.bag.admin.ch/bag/de/home/themen/mensch-gesundheit/chemikalien/nanotechnologie.html
The Netherlands	NanoNextNL	http://www.nanonextnl.nl/
UK	SAFENANO	http://www.safenano.org
**Nanosafety Initiatives in Asia**		
**Country**	**Initiative**	**Website**
China	Nanosafety Key Lab (CAS)	http://english.nanosafety.cas.cn/
China	National Center for Nanoscience and Technology (NCNST)	http://english.nanoctr.cas.cn/
Japan	Nano Safety Web Site (Japanese language only)	http://www.nanosafety.jp/
Singapore	Asia Nano Forum (ANF)	http://www.asia-anf.org/working-groups/nano-safety-risk-management/
Thailand	Nanosafety Information Center of Thailand (NICT) (Thai language only)	http://nict.sc.chula.ac.th/site/index.php

**Table 2:** Download numbers 2016 for various documents provided on the DaNa^2.0^-website (SOPs, literature checklist, Safety Data Sheets). These documents from our knowledge base are important sources for the safe development of new ENM/applications or products (statistical data collected by using the Webanalytics tool PIWIK, January–December 2016).

Documents	Downloads	% of Total Downloads
Overall download activities	6129	100%
DaNa SOP template	139	2%
SOPs from Projects	302	5%
Literature Criteria Checklist	149	2%
NanoCare Datasheets		
TiO_2_	3590	59%
ZrO_2_	403	7%
Carbon Black	256	4%
CeO_2_	248	4%
ZnO	226	4%
BaSO_4_	194	3%

## Appendix A

**Table A1:** Nanomaterials and applications included in the DaNa^2.0^ knowledge base [34].

Sector	Application in	Nanomaterials Involved	Estimated Exposure Potential Human	Estimated Exposure Potential Environment
Medical products & food	Bone cement Cancer therapy Contrast agent Dental prosthesis Dietary supplement Drugs Food additive Implants Pregnancy test Rapid diagnostics Vaccination Wound dressing	Barium sulphate Cellulose Gold Iron and Iron oxides Silicon dioxide Silver Titanium dioxide Zirconium dioxide Zeolite	High (exposure intended)	Medium (mainly via waste water)
Electronic goods	Color television tube Display Electrode Electronics LED Nano Wires Photovoltaic cell Processor RAM (Random-Access Memory) Touch screens	Carbon Nanotubes (CNT) Copper and copper oxides Diamond Gold Indium tin oxide (ITO) Quantum Dots Strontium carbonate Titanium dioxide	Low	Low (medium at end-of-life)
Construction and building	Abrasive and polishing agents Anti-fogging agents Cement Cobblestones Glass Glue Heated coatings Lacquer and plastics additive Lightweight construction Rubber Tools	Aluminum oxides Carbon Black Carbon Nanotubes (CNT) Cerium dioxide Diamond Gold Indium tin oxide (ITO) Silicon dioxide Titanium dioxide Tungsten carbide Tungsten carbide-Cobalt Zinc oxide Zirconium dioxide	Medium to low	low
House hold	Anti-fogging agents Cat litter Cleaning agent PET bottles Soft Toy Sports equipment Tennis racket Textiles Wallpaper	Carbon Nanotubes (CNT) Fullerenes Graphene Nanoclays Silicon dioxide Silver Titanium dioxide Titanium nitride Zeolite Zinc oxide	High to medium	Medium
Personal care products	Cosmetics Skin care Soap Suncream Tooth paste	Carbon Black Fullerenes Silicon dioxide Silver Titanium dioxide Zinc oxide	High (exposure intended)	Medium (mainly via waste water)
Pigments	Black pigments Facade and wall color Printing inkToner	Carbon Black Iron and Iron oxides Silicon dioxide Silver Titanium dioxide	High (when exposure intended, e.g., Tattoos) Medium for others	Medium to low (mainly via waste)
Automotive sector	Catalytic exhaust converter Diesel additive Fuel cell Lubricant Tires	Barium sulphate Carbon Black Cerium dioxide Copper and copper oxides Fullerenes Gold Platinum Silicon dioxide Zirconium dioxide	High to medium	High to medium
Agriculture and environmental sector	Environmental remediation Fertilizer Filtration Water softener Water treatment Wood preservatives	Aluminum oxides Copper and copper oxides Diamond Gold Iron and Iron oxides Zeolite	Medium to low	High to medium
Miscellaneous	Chemical catalyst Coating Film Pyrotechnics	Gold NanoClays Silicon dioxide Silver Strontium carbonate Titanium dioxide Zeolite	Low (Event related)	Medium to low
